# Topologically Optimized Anthropomorphic Prosthetic Limb: Finite Element Analysis and Mechanical Evaluation Using Plantogram-Derived Foot Pressure Data

**DOI:** 10.3390/biomimetics10050261

**Published:** 2025-04-24

**Authors:** Ioannis Filippos Kyriakidis, Nikolaos Kladovasilakis, Marios Gavriilopoulos, Dimitrios Tzetzis, Eleftheria Maria Pechlivani, Konstantinos Tsongas

**Affiliations:** 1 Advanced Materials and Manufacturing Technologies Laboratory, Department of Industrial Engineering and Management, School of Engineering, International Hellenic University, 57001 Thessaloniki, Greece; giankyri@iem.ihu.gr (I.F.K.);; 2 Information Technologies Institute/Centre for Research and Technology Hellas, 57001 Thessaloniki, Greece; nikoklad@iti.gr; 3 Digital Manufacturing and Materials Characterization Laboratory, School of Science and Technology, International Hellenic University, 57001 Thermi, Greece; d.tzetzis@ihu.edu.gr

**Keywords:** orthopedic biomechanics, anthropomorphic design, bioinspired topology optimization, mechanical properties, TPU, ground tire rubber composites, finite element analysis

## Abstract

The development of prosthetic limbs has benefited individuals who suffered amputations due to accidents or medical conditions. During the development of conventional prosthetics, several challenges have been observed regarding the functional limitations, the restricted degrees of freedom compared to an actual human limb, and the biocompatibility issues between the surface of the prosthetic limb and the human tissue or skin. These issues could result in mobility impairments due to failed mimicry of the actual stress distribution, causing discomfort, chronic pain, and tissue damage or possible infections. Especially in cases where underlying conditions exist, such as diabetes, possible trauma, or vascular disease, a failed adaptation of the prosthetic limb could lead to complete abandonment of the prosthetic part. To address these challenges, the insertion of topologically optimized parts with a biomimetic approach has allowed the optimization of the mimicry of the complex functionality behavior of the natural body parts, allowing the development of lightweight efficient anthropomorphic structures. This approach results in unified stress distribution, minimizing the practical limitations while also adding an aesthetic that aids in reducing any possible symptoms related to social anxiety and impaired social functioning. In this paper, the development of a novel anthropomorphic designed prosthetic foot with a novel Thermoplastic Polyurethane-based composite (TPU-Ground Tire Rubber 10 wt.%) was studied. The final designs contain advanced sustainable polymeric materials, gyroid lattice geometries, and Finite Element Analysis (FEA) for performance optimization. Initially, a static evaluation was conducted to replicate the phenomena at the standing process of a conventional replicated above-knee prosthetic. Furthermore, dynamic testing was conducted to assess the mechanical responses to high-intensity exercises (e.g., sprinting, jumping). The evaluation of the dynamic mechanical response of the prosthetic limb was compared to actual plantogram-derived foot pressure data during static phases (standing, light walking) and dynamic phenomena (sprinting, jumping) to address the optimal geometry and density, ensuring maximum compatibility. This innovative approach allows the development of tailored prosthetic limbs with optimal replication of the human motion patterns, resulting in improved patient outcomes and higher success rates. The proposed design presented hysteretic damping factor and energy absorption efficiency adequate for load handling of intense exercises (0.18 loss factor, 57% energy absorption efficiency) meaning that it is suitable for further research and possible upcycling.

## 1. Introduction

In recent years, a significant number of limb amputations have been reported for a variety of reasons, either health-related or traumatic amputations from accidents. In Western Europe, the elevated life expectancy has led to many cases of amputations due to neurological issues, infections, or chronic diseases such as diabetes. Meanwhile, in Eastern Asia, Australia, and North America, the majority of the reported cases refer to traumatic amputations due to road or workplace accidents. According to the National Institute of Health (NIH), approximately 15 new cases of amputations (health- or traumatic-related) are reported per 100,000 globally every year. In total, it is estimated that 57 million people out of the global population are living with limb amputations, and 75,000 prosthetic limbs are demanded each year for the new cases and the already reported ones. Notably, 69% of the reported cases involve lower limb amputations, especially foot and above-knee prosthetics [1,2,3,4]. The development of prosthetic limbs can help address kinematic impairments of the amputees, by enhancing mobility independence. This allows for broader involvement in social and physical activities, leading to improved quality of life and psychological well-being. It is also reported that the total replacement of the injured or infected part is the most effective way of assessing mobility issues, not only in terms of the mimicry accuracy of the human locomotion, but also in terms of safety, especially for problems occurring in the ankle region (Total Ankle Total Talus Replacement, TATTR) [5,6].

Research focused on the improvement of the efficacy of lower limb prosthetics has advanced over the last few decades, especially in material, design, and performance optimization. The traditional wooden frames have been replaced with advanced materials such as carbon fibers or stiff polymers. For the contact regions between the actual human tissue and skin, biocompatible polymers or metals are being utilized. For components that undergo surface contact with the ground, hyperelastic thermoplastics are being utilized with high energy absorption and damping capabilities. Material selection is an important aspect of the prosthetic limb synthesis process. The recent rejection rates for conventionally designed prosthetics are relatively high (rejection rates can reach up to 70%) and the majority of the developed prosthetics are deemed inadequate for actual use [7,8,9,10,11,12]. The development of anthropomorphic shapes for prosthetic parts has emerged as an end solution to the functionality issues regarding the reproduction of human locomotion. These designs allow for a better fit, stability, and balance, especially in cases of unilateral amputations, where the achievement of adequate symmetry and mobility is more challenging [13,14,15].

The optimization of the overall performance and the functional compatibility of the prosthetic compared to the actual human body part led to the insertion of novel processes for achieving better weight distribution across the structure, such as topology optimization (TO). With TO, strategic material removal is conducted with a density-based or truss-based approach to achieve the removal of excessive mass on the same bounding box. Especially in the truss-based approach, removal in repetitive patterns called unit cells is conducted (lattice geometries), to help provide the final structure-tailored mechanical properties. This method focuses on adjusting key microstructural parameters such as the size distribution, strut dimensions, cell shape, and density, with a variety of applications in the automotive, aerospace, and biomedical industry. Especially in the biomimetics field, the efficient exploitation of TO can lead to lightweight structures with enhanced strength-to-weight ratios that accurately mimic human locomotion and stress distribution patterns, leading to lower rejection rates for human prosthetics [16,17,18,19].

In the case of lower limb amputation prosthetics, an important issue that needs to be addressed is the achievement of unified stress distributions similar to a natural leg. This results in the minimization of discomfort and imbalance issues and enhances the durability of the final prosthetic. Especially in the ankle region, due to the direct contact with the ground, high damping and absorbing properties are demanded. The insertion of complex Triply Periodic Minimal Surface (TPMS) structures, such as gyroid or diamond, have been widely researched with the results showing that those structures are optimal for the development of materials with high elasticity or energy absorption. Therefore, the insertion of gyroid lattice geometries in the ankle region seems to be the go-to solution for the development of a novel topologically optimized prosthetic foot. Overall, the creation of efficient accurate prosthetics is a challenging aspect since each case demands tailored design and mechanical response. The insertion of TO, along with novel manufacturing techniques such as Additive Manufacturing (AM), which is known for its high precision and ability to mimic complex geometries, could stand as a reliable solution in the field of human prosthetics [20,21,22,23,24,25]. AM allows for the rapid customization and mimicry of complex geometries without the need for expensive molds. Combined with the appropriate Finite Element Analysis tools (FEA) for accurate computational investigation of the present static (standing) or dynamic phenomena (walking, running, and jumping), tailored designs can be manufactured. This can result in prosthetics with high precision, low rejection rates, and reduced material waste, leading to improved functionality, while simultaneously promoting financial and environmental sustainability [26,27,28,29].

**Figure 1:**
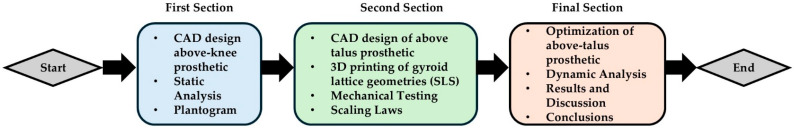
Flow chart of the research paper.

In the current study, the development of a novel prosthetic foot for TATTR with anthropomorphic design and the insertion of bioinspired gyroid lattice geometries was investigated. The insertion of TPMS lattices, and especially the gyroid geometry, allows for the enhancement of the energy absorption properties and shape-recovery properties which are vital for the accurate mimicry of joints, tendons, or ligaments. FEA and plantogram-derived foot pressure data were utilized to enhance the accuracy of load simulations during various movement phases (walking, running, and jumping). In the first section, a CAD design of a conventional above-knee prosthetic was followed by static analysis simulations to assess the forces that the lower above-talus region handles during standing. Then, an anthropomorphic lower prosthetic limb was designed with the aid of 3D scanning from an actual human foot. In the second section, an investigation of the optimal relative density of the proposed gyroid structure was conducted for the case of a hyperelastic thermoplastic composite material (TPU), with the aid of Selective Laser Sintering (SLS) AM. The 3D printed unit cells went under uniaxial compressive loads to assess the minimum adequate relative density for the development of the prosthetic foot in correlation with the maximum stress derived from the plantogram and the calculation of the structure’s scaling laws. In the third section, the evaluation of the structural integrity and the response of the proposed final design was conducted with the aid of Finite Element Analysis. The experimental results showed that the application of a hyperelastic TPU-based material on a gyroid lattice geometry design positively influenced the damping capabilities of the final design, allowing for unified stress distributions aligned with the actual plantogram data, making it adequate for possible manufacturing. The workflow of this study is briefly illustrated in Figure 1.

## 2. Materials and Methods

### 2.1. Initial Anthropomorphic Design

**Figure 2:**
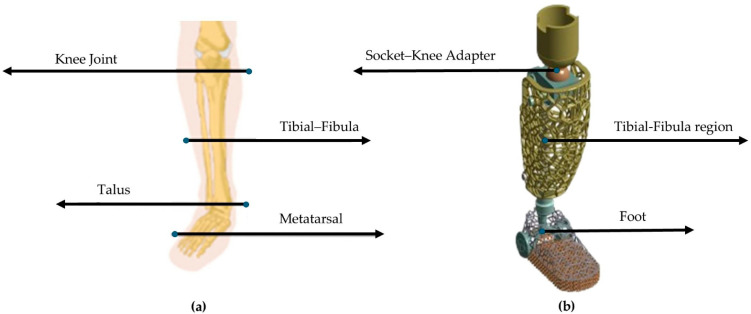
Conceptualization of the above-knee prosthetic design: (**a**) Human anatomy of leg region [8] and (**b**) Derived above-knee prosthetic for static integrity evaluation.

**Figure 3:**
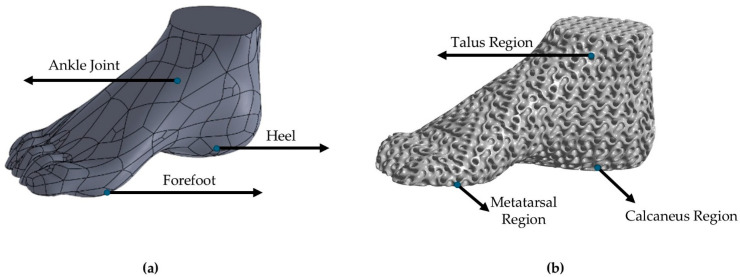
Conceptualization of the above-talus prosthetic design: (**a**) 3D scanned frame of an actual 10.5 US shoe size human foot and (**b**) Proposed anthropomorphic design with bioinspired lattice geometries.

For the initial anthropomorphic design conceptualization, an above-knee prosthetic with a conventional above-talus region was constructed, and an assessment of the static behavior was conducted. The simulation replicated the effect of the forces from standing on the lower limbs of an average human (75 kg). Following the static analysis, a dynamic approach to the above-talus prosthetic was conducted. The mimicry of the real human above-talus shape was advised to be the optimal design for the efficient stresses’ distribution on the final structure. Pitkin developed a mathematical model to describe the similarity rate of the developed prosthetic with a natural human leg called Index of Anthropomorphicity (IA). Results showed that a human-based design presented 25% reduced maximum loads in the ankle region compared to 3 non-anthropomorphic designs [30]. Therefore, for the design of the ankle prosthetic, a 3D scan of an actual male foot was conducted with the aid of an Artec Eva 3D scanner (Artec, Luxembourg, Luxembourg) and the raw data were converted to a compatible 3D CAD design file. The preliminary design was then inserted into Solidworks™ Version PDM 2023 (Dassault Systemes SE, Paris, Vélizy-Villacoublay, France) to eliminate rough edges and harsh geometries. Then, with the aid of nTopology™ Version 5.9.2 (nTopology Inc., New York, NY, USA), two different relative densities of the gyroid lattice geometry were inserted. A relative density of 60% represents the maximum relative density, the upper limit at which the proposed structure retains the characteristics of architected materials, according to the equivalent scaling laws. In contrast, structures with a relative density below 20% are challenging to manufacture and are classified as foam structures [31]. The final optimized design was evaluated with the aid of ANSYS™ Version 2024 R2 (ANSYS, Inc., Canonsburg, PA, USA) in loads reflecting hard fall conditions, such as jumping during a high-intensity sport, or during a stunt jump in free running. In Figure 2, the design conceptualization for the above-knee prosthetic is presented as aligned with the human leg anatomy, and in Figure 3, the above-talus prosthetic design conceptualization is presented as aligned with the CT-scanned human foot. Three main regions of interest were identified, the talus region which demands adequate shape memory elasticity to implicate the angular ankle movements, the metatarsal region which demands high elasticity and damping capabilities since it is the first contact region to the surface during high-intense-activity actions, and the calcaneus region which absorbs higher stresses during light activities such as walking. For standing and walking phenomena, the effective area was described as the summary of both the calcaneus and the metatarsal region, while in the dynamic response, only the metatarsal region was considered effective.

### 2.2. Materials

**Figure 4:**
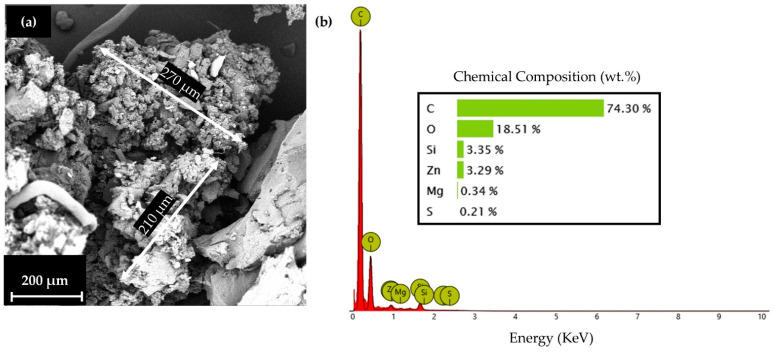
GTR powder characterization: (**a**) Particle shape and size analysis and (**b**) EDX analysis.

For the material selection, a Thermoplastic Polyurethane matrix, with carbon black traces for increased strength, was selected in powder form (Sinterit™, Krakow, Poland). It was reinforced with reacquired Ground Tire Rubber (GTR) [32,33] from RETIRE ABEE, Drama, Greece, which was shredded from end-of-life automotive tires into small pieces, along with the removal of the steel wires with the aid of magnets, and then the final milling of the rubber was made into a fine powder form, compatible with the matrix material. The blend was conducted in previous research with gradual mixing. The final blend consisted of 10 wt. % GTR. In Figure 4, an indicative particle morphology and size, as observed with the aid of Phenom ProX (Thermo Fisher Scientific, Waltham, MA, USA) Scanning Electron Microscope (SEM), is presented (Figure 4a), along with the EDX analysis of GTR (Figure 4b).

### 2.3. Additive Manufacturing

For the mechanical evaluation of the proposed lattice geometry and the identification of the optimal relative density (r.d.), in terms of lightweight design and structural integrity, different cubic sandwich structures in 3 different relative densities (20%, 30%, and 40% r.d.) with 30 × 30 × 30 mm dimensions were produced with the aid of Selective Laser Sintering (SLS) Additive Manufacturing (AM). The printing of the aforementioned structures was conducted with the aid of a Sinterit Lisa (Sinterit™, Krakow, Poland) 3D printer. The Sinterit Lisa 3D printer has an IR laser–diode source, and the laser beam power was set at 5 W. The scan speed was set at 100 mm/s, the layer height was set at 0.075 mm for optimal accuracy, and the printing temperature was around 100 °C, slightly below the melting temperature of both the reinforcement (GTR, 120 °C melting point) and the matrix (TPU, 160 °C melting point). The hatching distance was set at 0.2 mm and the laser beam diameter was 0.4 mm. The effect of GTR on TPU has been studied and the main properties are presented in Table 1.

From the data of Table 1, it is visible that the GTR insertion in TPU positively influences the vibration isolation properties, which are vital for the development of a prosthetic foot due to repetitive contact with the ground. Although the GTR insertion results in a structure with better elasticity and damping capability, high concentrations of GTR would result in excessive damping, leading to performance limitations. Also, high concentrations of GTR could lead to diminishing of the shape memory properties due to the elevation of the shape recovery time. This could lead to malfunctions in dynamic phenomena. The main boundary condition of functionality was set to be the loss factor. For the case of functional prosthetics or footwear, the appropriate range was 0.1–0.2. Therefore, GTR weight fractions higher than 20 wt.% were not studied for this case, and the optimal composition selected for this case was proved to be the one with the 10 wt.% GTR.

**Figure 5:**
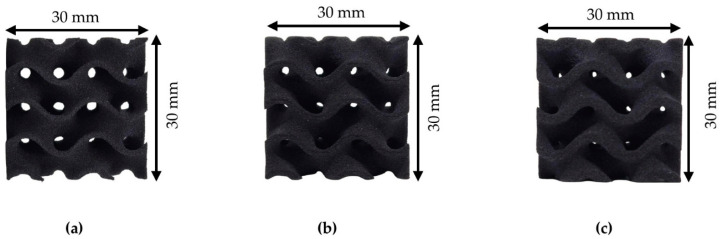
As-printed gyroid cubic unit cells (30 × 30 × 30) for: (**a**) 20% relative density; (**b**) 30% relative density; and (**c**) 40% relative density.

The layer height was set at the lower potential layer height range of the printer, at 0.075 mm, to ensure the optimal mimicry of the designed walls of the sandwich’s structures even at low relative densities and minimization of printing defects. At this point, it is worth mentioning that the SLS AM anisotropies are minimal (less than 10% difference in the mechanical integrity in each direction) due to the fusion process between the powder particles, which results in more unified structures and results. For the achievement of the maximum strength through the testing process, the loads were applied on the z-axis to avoid possible reduced overall response due to layer detachment phenomena. In Figure 5, the manufactured specimens are presented for each relative density.

### 2.4. Plantogram Foot Pressure Data

The plantogram experimental data that were used for this case were of a male basketball athlete with a total body mass of 75 kg, the average male adult body mass that is inserted in the evaluation of prototype structures. Data were recorded during the standing phase, the walking phase, and the landing phase of a standing vertical jump to assess the maximum applied stresses in the aforementioned situations. The calculation of the experimental data was conducted with the aid of the orthopedic insole manufacturing company Peditech (Peditech, Thessaloniki, Greece). The software-derived results from the plantogram were also compared to a lab-developed mathematical model. From the plantogram data, the calculation of the boundary conditions was conducted for the final optimized model. The average body mass used in the model was set also at 75 kg. The calculation of the total maximum load that a human foot handles during a hard fall is expressed by the following equation:

(1) $$F_{total}=F_{impact}+W_{human}=m\cdot \frac{dV}{dt}+W_{human}\;N$$

where *W_human_* is the total weight of the average person and *F_impact_* is the average force that comes off the kinematic motion of the foot during the landing process. The calculation of this force depends on the actual velocity of impact (*dV*) and the time of landing, *dt*. According to the existing literature, the average landing speed is around 3 m/s in cases of vertical jumps with existing momentum that happen in sports like basketball or volleyball or for landings from higher surfaces in extreme cases like free running. The average landing time for a hard fall is around 0.05 s. Therefore, the average calculated force from the mathematical model for hard fall situations has been estimated to be around 5300 N, which is around 7 times the human body weight [34,35,36].

### 2.5. Compression and Compression Cycling Mechanical Testing

The compression and the loading/unloading testing were conducted with the aid of the Testometric M500-50AT (Testometric Company, Rochdale, UK), which is equipped with a 50 kN load unit cell in alignment with the ISO 7433 standards [37]. For the compression testing, the cross-head speed was set at 10 mm/min, while for the loading-unloading/testing, the strain rate was also 10 mm/min. The energy absorption capabilities of the different structures were identified by the calculation of the overall Energy Absorption (EA) value. This value represents the Specific Energy Absorption per Volume unit (SEA_v_). The mathematical equation for the quantification of the EA capabilities describes the area beneath the stress–strain curve of an energy absorber and is expressed by the integral of the stress function in relation to the equivalent strain until the densification point [38] (Equation (2)):

(2) $$EA\left(d\right)={SEA}_{v}\left(d\right)=\int_{0}^{\varepsilon }\sigma \left(\varepsilon \right)d\varepsilon$$

In the aforementioned function, the term σ represents the observed compression stress at the equivalent experimental strain point (ε). Besides the representative EA value per volume unit, the EA capability can be also calculated per mass unit. This value is representative of the equivalent property-to-weight ratio. The insertion of lattice geometries greatly influences the strength-to-weight ratio of the final structure. The Specific Energy Absorption per Mass unit (SEA_m_) is calculated by the following equation:

(3) $${SEA}_{m}\left(d\right)=\frac{EA\left(d\right)}{p}=\frac{{SEA}_{v}\left(d\right)}{p}$$

In the aforementioned function, the term EA(d) stands for the overall energy absorption during the experimental process, as defined by Equation (2), and *p* stands for the density of the used material. To identify the overall efficiency of the absorber, the η(ε_α_) value is calculated with the aid of Equation (4):

(4) $$\eta (\varepsilon_{\alpha })=\frac{\int_{0}^{\varepsilon_{\alpha }}\sigma \left(\varepsilon \right)d\varepsilon }{\sigma_{\alpha }(\varepsilon )\cdot \varepsilon_{\alpha }}$$

In the aforementioned equation, the ratio between the area under the compression stress–strain curve which represents the actual EA capability of the structure on the particular experimental strain point (ε_α_), and the capability of the ideal absorber is expressed by the multiplication of the equivalent stress (σ_α_(ε)) at the experimental strain point (ε_α_), which is the last critical point of the curve where some oscillatory behavior is observed. This point is usually aligned with the densification point [31,39].

Besides the energy absorption capabilities, the evaluation of the compressive properties of the proposed lattice geometry allowed for the calculation of the tailored ideal relative density of the novel prosthetic limb, extracted by the calculation of the different scaling laws. The size effect is calculated using Equation (5):

(5) $$\frac{\Phi_{lattice}}{\Phi_{solid}}=c\cdot {\left(p_{relative}\right)}^{n}$$

where *Φ* is the equivalent property (e.g., the elastic modulus), *p* is the relative density of the lattice, and the variables *c* and *n* are constants of the equation that are dependent on the feedstock material. Therefore, an accurate estimation of the optimal relative density of the proposed architected lower limb prosthetic was allowed to confirm the final model characteristics [16].

### 2.6. Topological Optimization

**Figure 6:**
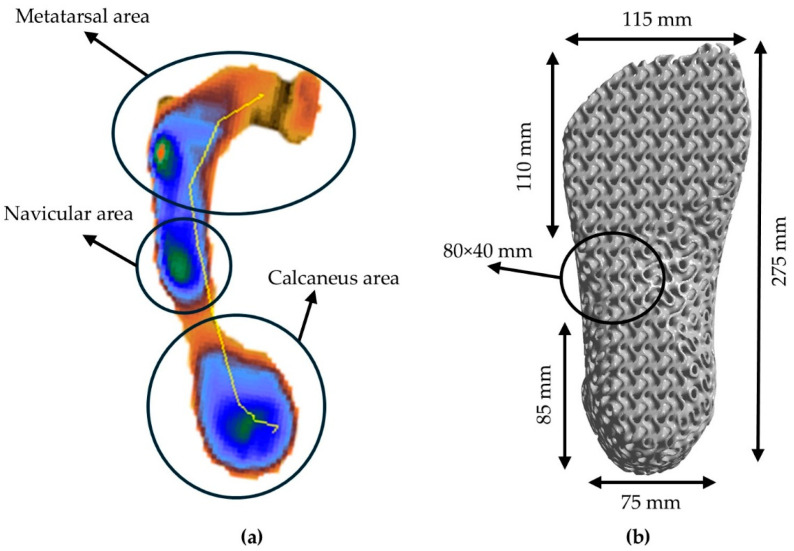
Conceptualization of contact surfaces: (**a**) Identified contact surfaces from patient-derived data and (**b**) Final dimensions of contact surfaces on the prosthetic design.

For the topological optimization of the proposed above-talus prosthetic limb, the nTopology™ Version 5.9.2. (nTopology Inc., New York, NY, USA) was utilized. The shape of the structure was designed in alignment with the Regions of Interest that were extracted from the plantogram contours. For the evaluation of the optimal lattice geometry parameters, the optimal solution in terms of weight and functionality was identified according to the equivalent scaling laws described in Equation (5). For the evaluation of the scaling laws, 3 different relative densities were tested (20% r.d., 30% r.d., and 40% r.d.). As mentioned, the proposed lattice geometry was the TPMS gyroid structure. TPMS-based structures present the greatest energy absorption and damping efficiency, which are key factors for the overall integrity of lower limb human prosthetics [16]. In Figure 6, the plantogram contours while standing (a), and the final proposed design with the metatarsal and calcaneus area dimensions (b) are presented, with a focus on creating an adequate landing area (metatarsal and calcaneus) to provide stability.

### 2.7. Finite Element Analysis

For the setup of the computational investigation of the proposed prosthetic limbs, the ANSYS™ Version 2024 R2 (ANSYS, Inc., Canonsburg, PA, USA) software was utilized. The developed models were inserted into an explicit dynamics model that accurately simulated the landing process after a hard fall. For the material model, experimental identification of the mechanical response and the properties of the microstructure was already conducted. The parameters were inserted in the Material Designer tool in ANSYS™ Version 2024 R2 and a Representative Volume Element (RVE) of the proposed composite (TPU-GTR 10 wt.%) was constructed. Since similar properties were presented regardless of the direction (x, y, and z) the isotropic elasticity model was utilized for the final structure. Regarding the boundary conditions, the initial velocity was calculated in Section 2.4 and was confirmed with the actual plantogram data (3000 mm/s), and the time of the experiment was set at 0.05 s, representing the actual conditions of a normal vertical landing. The applied load on the foot during the landing is located in the lower part of the foot, during the contact with the surface (Vertical Ground Force Reaction).

## 3. Results

### 3.1. Results of the Preliminary Design

#### 3.1.1. FEA Results

**Figure 7:**
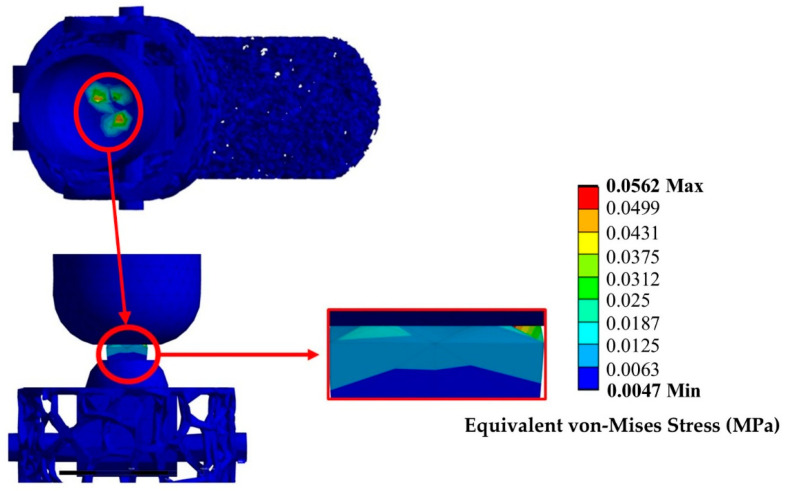
Equivalent stress distribution on the above-knee structure.

For the preliminary design, an above-knee prosthetic was constructed according to the literature and a static analysis was conducted to estimate stress concentrations on the lower limbs of a human being while standing. The results of the static analysis are presented in Figure 7.

The appearance of stress concentrations on the knee articulation is normal since the higher loads during standing are presented in the knee region and the lower back region above the hip. The maximum handled stress of around 56 kPa is aligned with the expected stresses of 40–100 kPa during standing. Therefore, the preliminary above-talus component presents adequate structural integrity for standing conditions and is eligible for further optimization. The main issue with the existing design is its complexity since three separate parts are involved, with one that must robotically mimic human locomotion (AI tools for neuronic movement mimicry are required). This complexity leads to elevated costs, and potential stress risks at the joint due to the highly elastic movements of the foot frame, particularly during dynamic shock loads from hard falls (e.g., vertical jumping, free running jumping) or high-intensity activities (jogging, sprinting). Therefore, a need for further optimization of the above-talus is essential and an assessment of the dynamic response was demanded since the ankle region experiences more loads during high-intensity activities. The optimized design aims to utilize the shape memory and elasticity material properties of TPU and GTR. This enables the accurate mimicry of human locomotion without the need for complex joints and advanced robotic technologies. The proposed design’s flexibility allows the use of it in cases of above-talus amputations as standalone prosthetics and in above-knee amputations as a component of a more complex structure. In cases of complex structures, the development of a unified prosthetic foot improves the connectivity with the lower adapter that implicates the tibia and fibula bone of the human leg, resulting in a more accurate mimicry of the actual human anatomy, with aesthetic and functional benefits.

#### 3.1.2. Plantogram-Derived Results

For the assessment of the boundary conditions of the dynamic phenomena that the ankle region handles, the results from an actual plantogram during mild exercise (walking, running) or intense exercise (e.g., jumping) were extracted. The results from the plantogram data are presented in Table 2.

The plantogram-derived results are presented as a reference point and as design guidance. The standing vertical jump in a training lab environment differs from an actual dynamic vertical jump, but a specific pattern was recognized. During kinematic exercises, there is a significant time when the maximum load is applied in the forefoot area described as the metatarsal zone. In Table 3, the overall specifications of the designed prosthetic foot are presented.

According to Equation (1), the maximum applied load during a highly intense action is 5300 N. The calculated maximum stress demands are extracted from Equation (6):

(6) $$P_{max}=\frac{F_{max}}{A}=\frac{5300}{0.0126}=420,700\;Pa=0.42\;MPa$$

### 3.2. Optimization Process-Insertion of Bioinspired Lattice Structures

#### 3.2.1. Compression Test Results

**Figure 8:**
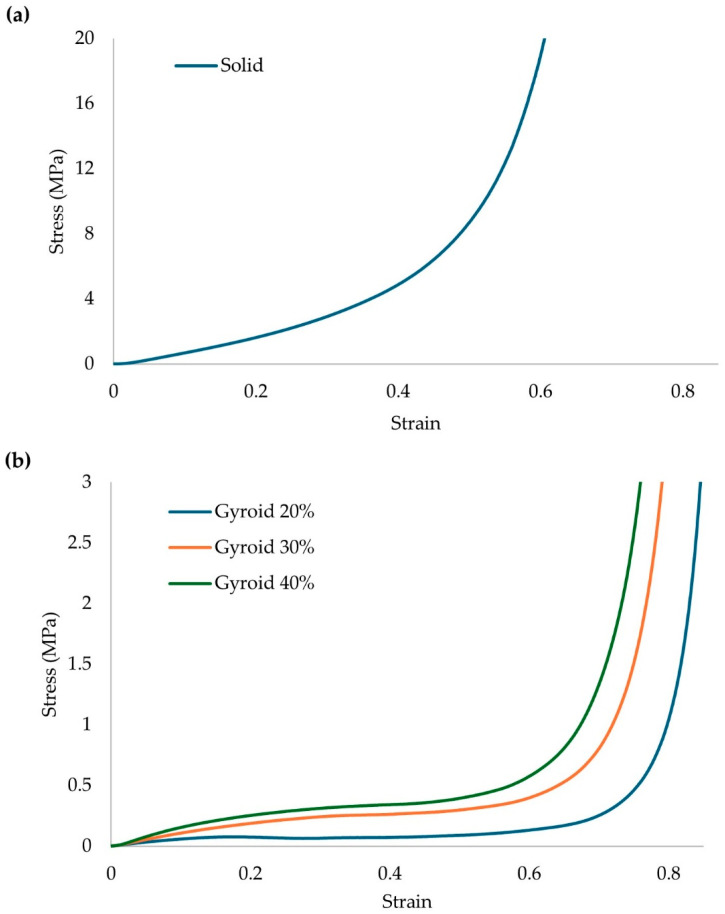
Compression results for: (**a**) Solid Structure and (**b**) Lattice structures TPU/GTR composites.

The results of the compression testing for the different relative densities of the proposed gyroid architected material are presented in Figure 8.

From the results of Figure 8, it is visible that there is a significant drop in the overall strength of the structure with the insertion of lattice geometries. It is also visible that a proposed structure with 20% relative density would not be able to handle the compressive stresses of an actual human (0.42 MPa) during an intense activity. TPU/GTR composites present excellent shape recovery ability, but in loads higher than the densification point, the recovery time is relatively short, meaning that the structure will keep the deformed shape during repetitive loads in this range. This could lead to diminishing absorption properties and leading to discomfort in other joints of the human body (knees, hips) resulting in possible injuries. In Table 4, the main mechanical properties are presented for the solid structure and the structures with lattice geometries.

In Table 5, the energy absorption properties (overall EA, EA per unit mass, overall efficiency) for the solid and the gyroid structures are presented.

#### 3.2.2. Identification of the Scaling Laws

**Figure 9:**
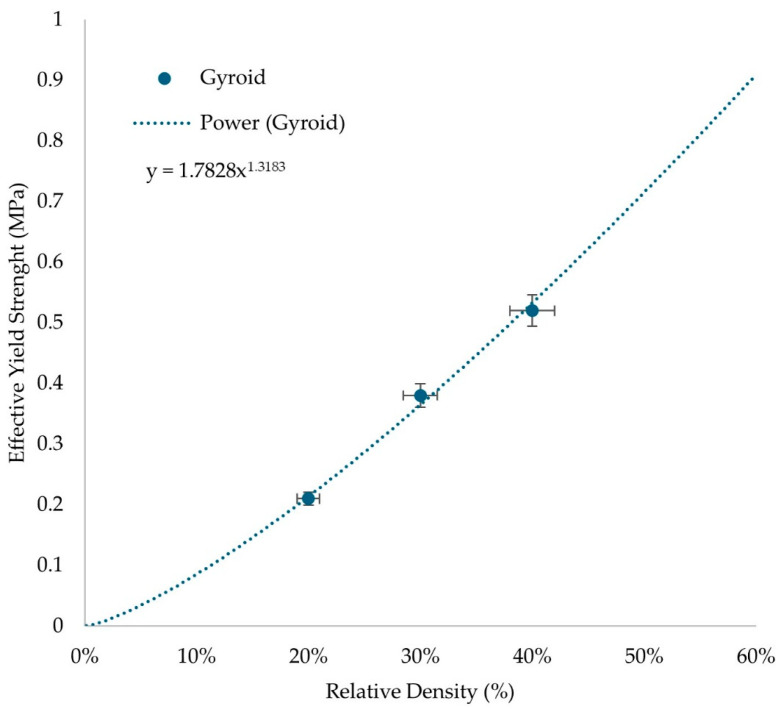
Scaling laws identification of the gyroid lattice geometry of TPU-GTR matrix material.

The results from Table 4, were used to describe the distinct behavior of the compressive stress at the densification point of the gyroid structure. With the aid of Equation (5), the main constants that describe the scaling laws for the proposed structure were extracted. Those constants are material and lattice geometry dependent. The results are presented in Figure 9.

According to Equation (6), and from the results of Figure 9, the c and n constants of the scaling law equation can be identified, and the power of the trendline function is the n constant which is around 1.32. This price is in the normal range for elastomeric thermoplastics. This factor indicates a mild bending-dominated behavior which is an ability that hyperelastic materials possess. The constant of the trendline is equal to Φ_solid_·c, therefore, the c constant in this situation is 0.37. Therefore, from the utilization of Equation (6) the minimum relative density for the development of an architected material that can stand the maximum calculated stresses of 0.42 MPa is 33%.

#### 3.2.3. Loading/Unloading Results

**Figure 10:**
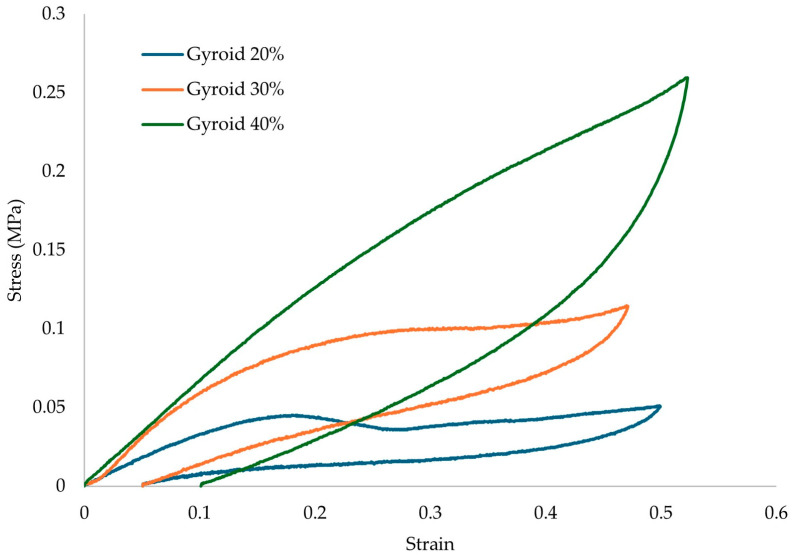
Hysteresis loops from loading/unloading tests under compression.

Compression loading/unloading testing was conducted to identify the structure’s damping capabilities. The loading conditions were conducted till the 60% point of the densification point region. The results of the loading/unloading experiments for the lattice structures geometries are presented in Figure 10 and the absolute damping values of the proposed structures are presented in Table 6.

From the results in Table 6, it is visible that the damping factor is not geometry dependent and the only factor that determines this property is the matrix material selection. Since in all cases the same matrix was selected, the damping capability remained almost identical. The final absolute value of approximately 0.18 is adequate for the development of a lower prosthetic since it is similar to the actual range of damping capabilities of sports footwear (0.1–0.2). Values below 0.1 could lead to high discomfort and injuries in other tendons or joints due to the significant elevation of the number of oscillations in the structure. Values above 0.2 could lead to a structure with high energy losses, therefore reducing the performance of the user and also resulting in irregular work production during the movement compared to the actual human body part. This could lead to muscular atrophies on the side where the prosthetic is attached.

### 3.3. Optimized Model Results

After identifying the minimum relative density in terms of functionality, a prosthetic foot with 40% relative density was constructed and went under explicit dynamic loads. The 40% R.D. was selected after taking into consideration the minimum calculated R.D. from the scaling laws section, along with some assumptions regarding possible deviations in order to minimize the chances of material failure. The boundary conditions were thoroughly described in Section 2.7 and Section 3.2. In Table 7, the main elastic properties (Young’s Modulus E, Shear Modulus G, and Poisson’s ratio v) of the material are presented in the x, y, and z directions, as extracted from the Material Designer.

**Figure 11:**
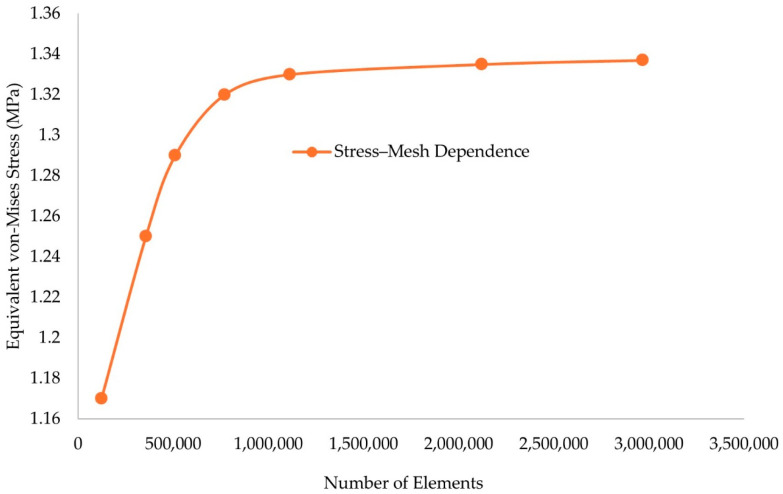
Mesh convergence analysis results for the prosthetic foot model.

From Table 7, it is visible that the aforementioned material presented similar mechanical properties regardless of the axis of the applied load. Therefore, the isotropic elasticity model was utilized to describe the material used for the development of the human prosthetic model. In Figure 11, the mesh convergence study results are presented for the final prosthetic foot model.

**Figure 12:**
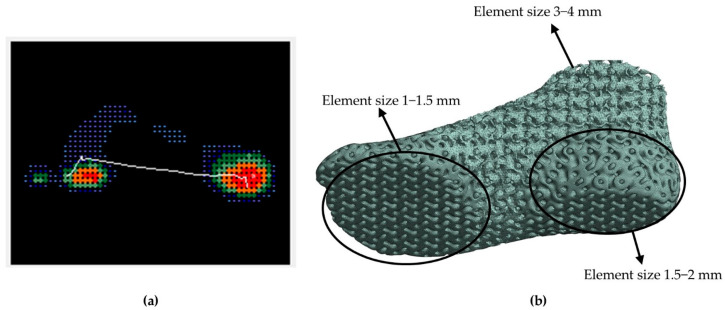
Mesh generation process: (**a**) ROI from plantogram contours and (**b**) Final mesh concentrations in ROI.

In alignment with the plantogram contours, the ROI that were identified had finer mesh with an element size 2–3 times smaller than the non-contact regions. This element ratio was kept similar in all cases. The results in Figure 11 confirm that with this element size distribution analogies, mesh independent results occur with a mesh of around 900,000 to 1,100,000 elements. A finer mesh was used in the heel region around the calcaneus bone, at the metatarsal bones zone, and at the equivalent contact regions on the surface, taking into consideration the plantogram contours. The element type for the selected model was SOLID 187, which is the adequate mesh for irregular shapes and complex geometries such as lattice structures consisting of hyperelastic materials; the final element size ranged from 1 to 4 mm (around 1 mm in the ROI of the lower foot). In Figure 12, the mesh generation process is presented, the ROI was identified by the plantogram contours and then transmitted into fine mesh areas and coarse mesh areas, as shown.

**Figure 13:**
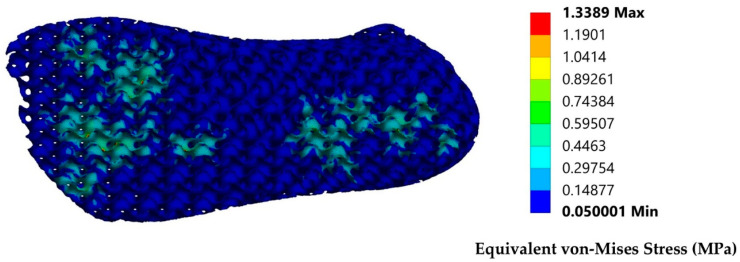
Stress distribution after the simulation of a landing process from high-intensity activity.

In Figure 13, the equivalent stress contours are presented after dynamic testing.

**Figure 14:**
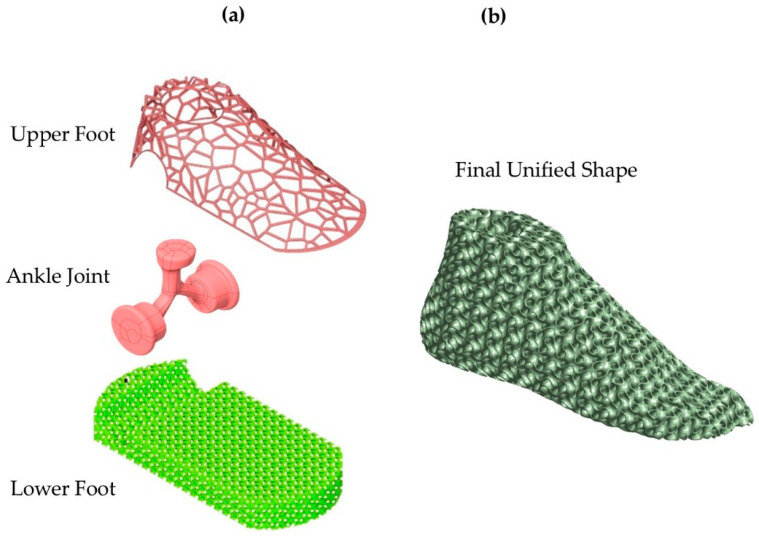
Final improvement of the above-talus component: (**a**) Preliminary components and (**b**) The final unified design.

From the results in Figure 13, it is visible that the main stress concentrations are happening in the metatarsal area and some in the heel area. The maximum values are detected in the metatarsal area with the range being between 0.29 and 0.44 MPa. This average value is similar to the 0.35 value that was calculated by the plantogram results. The proposed relative density of 40% presented a maximum tolerated strain before the densification of 0.52 MPa, therefore meaning that the final structure can present a FOS of around 1.5, which is described as ok, meaning that this structure could tolerate forces of around 7500–8000 N. Therefore, the proposed prosthetic could work as a standalone prosthetic for the above-talus amputations or as a part of a more complex above-knee prosthetic. Those values are similar to the actual maximum human foot tolerance in vertical loads during landing. In both cases, the development of a unified structure has significant advantages in terms of safety, comfort, and cost because no complex mechanisms have been developed. The ankle region is a region with high elastic and angular moves, and the existence of a robotic joint in this region could risk possible prosthetic failure in moves that could lead to high deformations. Therefore, the introduction of a hyperelastic architected material could lead to full functionality by utilizing its shape memory properties. The alteration of the talus component is presented in Figure 14.

**Figure 15:**
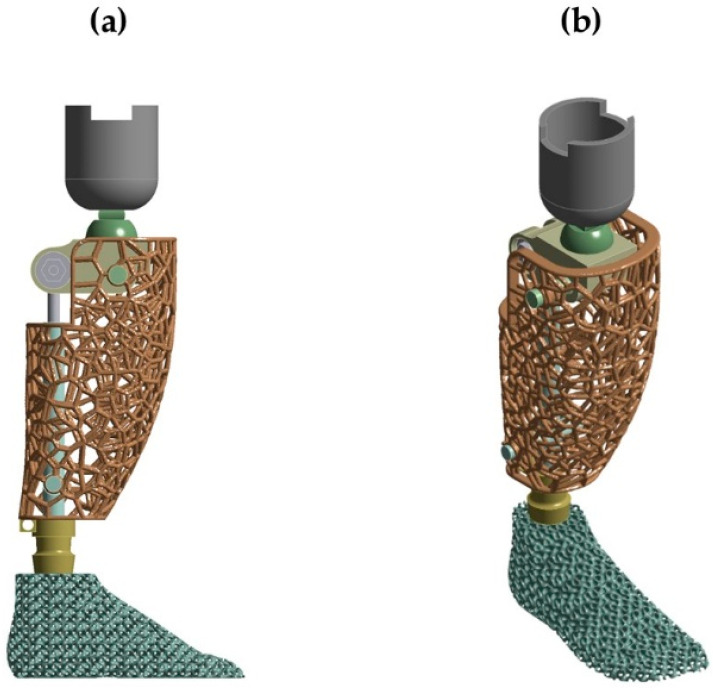
Suitability of the proposed prosthetic on a complex above-knee prosthetic structure: (**a**) Side view and (**b**) Isometric view.

The final component can be embedded with the other components of the prosthetic limb with thermal bonding processes. An illustration of the final prosthetic foot design in a complex above-knee prosthetic, where thermal bonding processes between the prosthetic foot and the axle were applied, is presented in Figure 15.

According to the ISO 10328:2016, for the evaluation of the structural integrity of the proposed structure, the minimum load tolerance of an elastomeric structure has to be two times the human body weight. In this case the minimum required tolerance is 1500 N which is, and according to ISO 22675:2024 for the dynamic testing of prosthetic feet and ankles, the final structure must be able to tolerate at least 60–80% of the maximum peak static force of 1500 N, calculated at around 900–1200 N for a repetitive number of cycles [40,41]. Possible future research can conclude the component-by-component manufacturing of an actual prototype and the dynamic testing and fatigue analysis according to the aforementioned ISOs for the ankle component.

## 4. Conclusions

The presented study focuses on the assessment of the static and dynamic behavior of prosthetic limbs for leg amputations. The objective of this research was to assess the structural integrity and functionality of the proposed lower limb prosthetics using actual data from mild- and high-intensity activities, extracted from plantogram studies in athletes. The incorporation of an anthropomorphic design along with the utilization of the shape memory capabilities of TPU and GTR materials allows for the development of novel prosthetics without multiple components, complex joints, or the demand for AI tools or soft robotics. The optimal geometry and relative density for the proposed design proved to be 40%. In this density, significant tolerance capability of the maximum applied forces was observed, along with unified stress concentrations in the metatarsal and calcaneus regions, aligned with the human foot’s landing mechanics. The FEA contours illustrated that the final design was able to precisely mimic both static and dynamic loads. The damping factor of 0.18 is within the appropriate range of 0.1–0.2 and ensures the elimination of potential discomforts. This loss factor value also allows for the adequate production of work during the movement, similar to the human ankle, leading to the minimization of possible irregular load distributions on other tendons or joints (knee, hips) and reducing the risk of possible muscle atrophies or injuries. Future research should focus on the development and manufacturing of an actual prototype with the utilization of 3D printing technology. It is important to state that AM technology is the most suitable for the precise development of patient-specific personalized designs such as those in the field of biomimetics. A possible assessment of the mechanical performance confirming the FEA results could be conducted by lab-scale experiments to assess the maximum compressive loads and the elastic capabilities of the proposed design. Tests such as fatigue analysis, impact testing, and cyclic loading are vital for possible upscaling. In the upcycling process, it is also vital to take into consideration that possible deviations could occur from nonlinearities related to the skin–socket interactions or due to the viscoelasticity of the human soft-tissue. This intermediate phase is vital before the development of a working prototype.

## Figures and Tables

**Table 1:** Material characterization of solid TPU-GTR structures.

Material	Compressive Strength at the Densification Point (MPa)	Damping (Loss) Factor (%)	Overall Energy Absorption (kJ/m^3^)
TPU	4.04 ± 0.5	15.1 ± 1.5	246 ± 21
TPU-GTR 10 wt.%	4.85 ± 0.5	18.4 ± 1.7	321 ± 27
TPU-GTR 20 wt.%	5.69 ± 0.6	21.0 ± 2.2	414 ± 32

**Table 2:** Plantogram-derived results.

Human Motion	Stress (MPa)	Effective Area (cm^2^)	Velocity (m/s)
Standing	0.044	229	0
Mild Exercise	0.245	201	1.02
Intense Exercise	0.349	125	3.11

**Table 3:** Prosthetic foot design specifications and areas of interest.

Surface	Max Length (cm)	Max Width (cm)	Effective Area (cm^2^)
Metatarsal (Forefoot)	11	11.5	126.5
Navicular (Middle foot)	8	3.5	28
Calcaneus (Heel)	8.5	7.5	63.75
Overall	27.5	7.5–11.5	220.25

**Table 4:** Main mechanical properties extracted from the compression tests.

Structure	Densification Point Stress (MPa)	Densification Point Strain (-)
Solid	4.85 ± 0.5	0.42 ± 0.04
Gyroid 20% r.d.	0.21 ± 0.02	0.71 ± 0.06
Gyroid 30% r.d.	0.38 ± 0.03	0.61 ± 0.05
Gyroid 40% r.d.	0.52 ± 0.04	0.59 ± 0.05

**Table 5:** Main energy absorption properties extracted from the compression tests.

Structure	SEA_v_ (kJ/m^3^)	SEA_m_ (kJ/kg)	η(ε_α_) (%)
Solid	320 ± 27	272 ± 23	41 ± 3
Gyroid 20% r.d.	11 ± 1	46 ± 4	59 ± 6
Gyroid 30% r.d.	34 ± 3	96 ± 8	58 ± 5
Gyroid 40% r.d.	44 ± 4	93 ± 7	57 ± 5

**Table 6:** Damping properties of the gyroid structures.

	Gyroid 20% r.d.	Gyroid 30% r.d.	Gyroid 40% r.d.	Solid
Damping factor (%)	17.8 ± 1.4	18.8 ± 1.6	17.6 ± 1.3	18.4 ± 1.7

**Table 7:** Elastic properties of the composites’ RVE unit cell.

Property Direction	Young’s Modulus (E), MPa	Shear Modulus (G), MPa	Poisson’s Ratio
X-axis	35.35	12.42	0.411
Y-axis	35.42	12.49	0.413
Z-axis	35.25	12.55	0.412

## Data Availability

Data sharing is not available.
